# Expression of concern: The extraction of essential oils from patchouli leaves (*Pogostemon cablin* Benth) using a microwave air-hydrodistillation method as a new green technique

**DOI:** 10.1039/c8ra90092b

**Published:** 2018-11-27

**Authors:** Andrew Shore

**Affiliations:** Royal Society of Chemistry Thomas Graham House, Science Park, Milton Road Cambridge CB4 0WF UK advances-rsc@rsc.org

## Abstract

Expression of concern for ‘The extraction of essential oils from patchouli leaves (*Pogostemon cablin* Benth) using a microwave air-hydrodistillation method as a new green technique’ by Heri Septya Kusuma *et al.*, *RSC Adv.*, 2017, **7**, 1336–1347.

*RSC Advances* is publishing this expression of concern in order to alert our readers that we are presently unsure of the reliability of the data reported in the article.

The Royal Society of Chemistry has been provided with credible information suggesting that the GCMS results presented in the paper may not be reliable. The majority of the microwave hydrodistillation (MHD) GCMS data presented in Table 5 has been reproduced from a previously published paper by the authors (*Periodica Polytechnica Chemical Engineering*, 2017, **61**, 82–92), which has not been cited in this *RSC Advances* paper. The reproducibility seen between the GCMS data in these two papers is very unlikely. The authors have been unable to provide raw GCMS data for the measurements presented in the *RSC Advances* article.

The Royal Society of Chemistry is currently seeking support from the affiliated institution in order to investigate this matter and establish whether the reported results are reliable. An expression of concern will continue to be associated with this manuscript until we receive information from the institution on this matter.

Andrew Shore

19th November 2018

Executive Editor, *RSC Advances*

## Supplementary Material

